# LTP after Stress: Up or Down?

**DOI:** 10.1155/2007/93202

**Published:** 2007-03-13

**Authors:** Marian Joëls, Harm J. Krugers

**Affiliations:** SILS-CNS, University of Amsterdam, 1098 SM Amsterdam, The Netherlands

## Abstract

When an organism is exposed to a stressful situation, corticosteroid levels in the brain rise. This rise has consequences for behavioral performance, including memory formation. Over the past decades, it has become clear that a rise in corticosteroid level is also accompanied by a reduction in hippocampal long-term potentiation (LTP). Recent studies, however, indicate that stress does not lead to a universal suppression of LTP. Many factors, including the type of stress, the phase of the stress response, the area of investigation, type of LTP, and the life history of the organism determine in which direction LTP will be changed.

When an organism is exposed to stress—here defined as any perceived internal or external disturbance of homeostasis—information about the stressful situation will reach an array of brain regions, including parts of the limbic system and areas involved in sensory processing [1]. The output from these areas funnels through the nucleus paraventricularis of the hypothalamus, where it can give rise to activation of two hormonal systems, that is, the rapid sympatho-adrenomedullar system and the slower-acting hypothalamo-pituitary-adrenal system (Figure 1). Activation of these systems leads to creased levels of adrenaline and corticosterone (cortisol in humans), respectively. These hormones not only affect peripheral organs but also feed back on the brain. Via intermediate steps, adrenaline can result in release of noradrenaline from central projections, in part to the very same areas that were involved in the initial processing of the stressful situation. Corticosterone feeds back at the level of the pituitary and hypothalamus, where it serves to normalize the release of stress hormones, so that approximately 2 hours after the initial stress exposure the release of corticosterone is restored to its prestress level (see Figure 2). Corticosterone, however, also reaches many extrahypothalamic regions. Those cells that carry receptors for the hormone will respond.

Studies over the past decades have shown that in brain corticosterone effects are mediated by two receptor types, that is, the mineralocorticoid receptor (MR) and the glucocorticoid receptor (GR) [1–3]. Both receptor types act as nuclear transcription factors, altering the transcription of specific sets of responsive genes, thus inducing slow but persistent changes in the protein content and hence function of cells [4]. MRs have a very high affinity for corticosterone and therefore will already be substantially occupied when the animal is observed under rest [1–3]. MRs have a restricted distribution, with high expression levels in all hippocampal subfields, the central amygdala, lateral septum, and some motor nuclei in the brain stem, but low levels in nearly all other parts of the brain. GRs, conversely, are widespread and encountered not only in neurons but also in glial cells. They have a relatively low affinity and will only become gradually activated when corticosteroid levels rise, such as occurs after stress exposure. The differential occupation ratio of MRs and GRs is particularly relevant to those cells that co-express both receptor types, such as principal cells in the CA1 region, the dentate gyrus, and the central amygdala. In these cells, receptor activation under physiological conditions will shuttle between predominant MR activation on the one hand and concurrent MR and GR activation on the other hand.

When it was realized that corticosteroids bind to receptors in brain [2, 5], people started to wonder how these hormones affect behavior and more specifically memory performance. In those days, it was also realized that long-term potentiation in limbic regions may play an essential role in the formation of memory [6], through an NMDA-receptor requiring mechanism [7]. Soon the first studies appeared describing the effect of stress on long-term potentiation and since then many more have been published.

The first observation was that behavioral stress, such as exposure to an inescapable shock, impairs LTP induction in the rat CA1 hippocampal area [8, 9]. This finding was corroborated in subsequent studies [10–12]; it was shown to involve the ERK pathway [13]. Subsequently, studies demonstrated that stress facilitates the induction of LTD [11, 14, 15], through a GR-requiring mechanism [16]. Even a short period of novelty suffices to shift the balance between LTP and LTD [15]. The reduction in LTP was also seen with administration of high doses of corticosterone either either in vivo [17] or in vitro [12, 18], indicating that corticosterone may be the leading hormone in the effects observed after stress. Optimal LTP induction was observed with low to moderate amounts of corticosterone [17]. In the absence of corticosterone, LTP induction was impaired, pointing to an inverted U-shaped dose dependency [17]. With respect to the falling limb of the inverted U-shape, an inverse relationship between the concentration of corticosterone and the ability to induce LTP was observed. This implies that severe stressors and/or stressors of longer duration particularly suppress the induction of LTP. A U-shaped dose dependency has also been described for corticosteroid effects on several single cell properties of CA1 pyramidal neurons, for example, the amplitude of voltage dependent Ca-currents, the cell firing frequency accommodation, and the responsiveness to serotonin [19, 20].

Exactly how stress or glucocorticoids suppress LTP and facilitate LTD is still not well understood. In vivo the phenomenon at least requires NMDA-receptor activation at the time of stress exposure [11] and an intact/active amygdala [21, 22], although all effects of corticosterone can be readily seen in vitro in a “reduced” hippocampal preparation, that is, in the absence of amygdala input [12]. It has been proposed that stress/glucocorticoids induce a variety of effects, including a change in the increase in the after-hyperpolarization amplitude [23–25], calcium current [26, 27], or LTP-like changes in glutamate transmission [28, 29], which all may interfere with the potential to subsequently evoke LTP [30, 31] in a metaplastic manner [32].

But does stress indeed universally impair LTP? No, so much has become clear over the past years. First, the balance between the various hormones that are released after stress exposure is very important. In some situations and in some individuals particular challenging situations may lead to more sympathetic drive relative to the HPA-axis or vice versa. As both noradrenaline (e.g., [33]) and corticotrophin releasing hormone [34] increase LTP, the abundance of these hormones relative to that of corticosterone is very important in determining the overall effect of stress.

Secondly, while it is generally agreed that LTP depending on NMDA-receptor activation is impaired by stress and corticosterone [11, 35], such impairment is not always seen for other forms of LTP. Thus, LTP that critically depends on voltage-dependent calcium channels (VDCC) is facilitated by the same dose of corticosterone that impairs the NMDA-type of LTP [36]. The facilitation of VDCC-type LTP involves activation of the GR. These observations may signify that behavioral paradigms that involve VDCC- (rather than NMDA-) type of LTP are promoted by prior stress exposure. This may, for example, be relevant for the formation of fear memory that was shown to involve VDCC-type of LTP in the amygdala [37].

A third factor that needs to be taken into account is the array of brain areas that are involved in a particular stress situation. Some stressors may involve activation of the amygdala, others not. Some stressors may activate brain stem regions involved in the processing of painful situations, others not, and so on. Not only do these areas differ with respect to their corticosteroid receptor expression patterns; but also cells that do express both MRs and GRs not always respond in the same way to an elevation in the level of the hormone [20]. For instance, both CA1 pyramidal neurons and granule cells in the DG highly express MRs as well as GRs. While corticosterone and stress consistently suppress the induction of CA1 LTP in vivo and in vitro, the outcome in the DG is less clear. Suppression of LTP was seen with very high corticosteroid concentrations [38] or tail shocks [39]. But in other instances, no effect was observed [40–42] or even an enhancement [43]. In this respect, it is important to note that cells in these various brain regions have specific properties and are incorporated in unique network constelations, so that even if corticosterone would evoke the same effect at the single cell level, this would not always result in the same effect on LTP. In the case of DG LTP, (indirect) input from the amygdala seems to play a crucial role [44, 45].

It is also very relevant to consider at which *stage* of the stress exposure effects on LTP are examined. The impairment of NMDA-type LTP always refers to the situation that stress and/or corticosterone are given some hours before the induction of LTP, allowing enough time for gene-mediated effects to develop. But recently, it was shown that corticosterone can also exert rapid nongenomic effects, via the MR [46]. These rapid effects result in an enhanced release probability of glutamate from Schaffer collateral terminals [46, 47]. In this way, stress may lead, in concert with noradrenaline and corticotrophin releasing hormone, to a facilitation of glutamate transmission, thus causing an endogeneous form of LTP. Moreover, it was found that through this rapid mode of action corticosterone can enhance LTP induced in the CA1 region by electrical stimulation, but only when the presence of corticosterone and the induction of LTP coincide [48]. Along the same line, it was found that LTP in the dentate gyrus is prolonged by stress through a nongenomic MR-mediated effect [49].

Finally, the response to a stressor or to corticosterone is also determined by the history of an organism. A well-documented example is the situation after chronic stress. It is extremely difficult to induce LTP in animals that have been exposed to repetitive stress in the weeks before the experiment [42, 50], even when corticosterone levels at the time of LTP induction are low to moderate, that is, at a level where normally LTP is readily evoked. When corticosteroid levels are then raised [42], LTP can still not be evoked, so that there seemingly is no effect of GR activation on LTP in animals with a history of chronic stress. A second example concerns the effect of maternal care. Preliminary observations indicate that animals which received very little maternal care have poor LTP when they are adult, as opposed to animals which received very much maternal care [51]. Interestingly, while LTP is suppressed by corticosterone in the latter group (as it is in the average population), it is enhanced in the former. This is reminiscent of behavioral studies in apolipoprotein E knockout mice, where corticosterone impaired spatial learning abilities in the wild types but improved behavioral performance in the knockout mice [52].

All in all, there is consensus that some hours after stress, LTP induced via NMDA receptors in the CA1 area is impaired, while LTD is facilitated (Figure 2). However, opposite effects on LTP can be found when the effects of stress are studied (i) at an earlier point in time, that is, when corticosteroid levels are still high; (ii) in other brain regions, for example, the dentate gyrus; or (iii) under conditions or in brain areas where VDCC-type of LTP is more prominent, for example, in the amygdala.

How could these effects of stress/corticosterone on LTP potentially affect memory formation? The prediction is that encoding of information which critically depends on the CA1 area is promoted by the concerted (nongenomic) actions of corticosterone, corticotrophin releasing hormone, and noradrenaline; this takes place during the initial phase of the stress response, that is, as long as the hormone levels are high. At the same time, a genomic cascade of events starts which through, for instance, enhancement of firing frequency accommodation and hyperpolarizing responses to serotonin as well as suppression of noradrenergic responses gradually leads to normalization of CA1 excitability. Part of this recovery process is also an enhanced threshold for LTP induction so that information reaching the same area some hours after the stressful event must be salient enough in order to overcome this heightened threshold, to be encoded. This will help to preserve the earlier encoded information. Both the initial phase (that promotes LTP and depends on catecholamines, peptides, and nongenomic MR actions) and the later “preserving” phase (involving genomic GR-mediated events that prevent LTP from being induced at that time) are assumed to be necessary for efficient consolidation of information.

This view is in line with most of the current data on the role of stress hormones in encoding of information. Behavioral studies in rodents indicate that MRs are more important in the initial (rapid) reaction to novelty and the acquisition of a learning task, reviewed in [53]. The more acute nature of these effects could be compatible with nongenomic actions, although this has not been investigated so far. In addition to these more rapid corticosteroid mediated effects, actions of other rapidly acting stress-related factors like noradrenaline and neuropeptides are of course also important for the encoding of information; for reviews see [54, 55]. For instance, behavioral studies in rodents have shown a very nice correlation between the amount of noradrenaline released in the basolateral amygdala and memory performance in an inhibitory avoidance task [56]. In addition to these aminergic effects and MR-dependent effects on reactivity, strategy, and acquisition, there is also ample evidence for a role of slow gene-mediated hormone effects in learning processes, as observed in several learning paradigms, including inhibitory avoidance behavior, spatial learning, and object recognition [57–60]. The consensus is that stress hormones released within the context of a learning task promote the consolidation of information [53]; this is different from the role of stress hormones in retrieval (not subject of this commentary, for review see [61]). Experimental evidence points to a critical role for GRs in these aspects of the learning process. First, selective GR agonists like RU 28362 are very effective in promoting the encoding of information [57]. Second, interference with DNA binding of GR homodimers prevents corticosterone from being effective in learning tasks [62]. In humans too, elevated levels of cortisol within the context of the learning situation are important for optimal memory performance [63].

The stronger the emotional value of the stressful situation is, the more other areas of the brain will become involved, in particular, the amygdala nuclei. In that case, the likelihood of facilitated LTP not only *during elevation* of corticosteroid levels but also *after normalization* of these levels increases. The delayed normalizing effect of corticosterone via a GR-dependent enhancement of cell firing frequency accommodation, stronger serotonergic hyperpolarization, and suppression of excitatory noradrenergic input then becomes essential to restrain the behavioral response to stress. The latter may be insufficient in individuals with a strong sympathetic drive but hypoactive HPA system, a situation often described for people susceptible to the development of posttraumatic stress disorder [64]. This could contribute to inadvertent engraining of a traumatic event and an inability to forget it.

The link between electrophysiological studies on LTP and behavioral observations is still tenuous. Ideally, one would like to study ongoing electrical activity and the possibility to induce LTP by tetanic stimulation in freely moving animals, that is, in behaviorally relevant situations. A complicating factor is that only part of the synapses part are implicated in synaptic strengthening during learning [65], so that advanced data acquisition and/or analyses methods are necessary to achieve the required spatial resolution. Clearly, such information with regard to the effects of stress on LTP, learning, and memory is presently not available. Studies, using these approaches and taking the nature, intensity, and phase of the stressor as well as the life history of the organism into account, are highly needed.

## Figures and Tables

**Figure 1:**
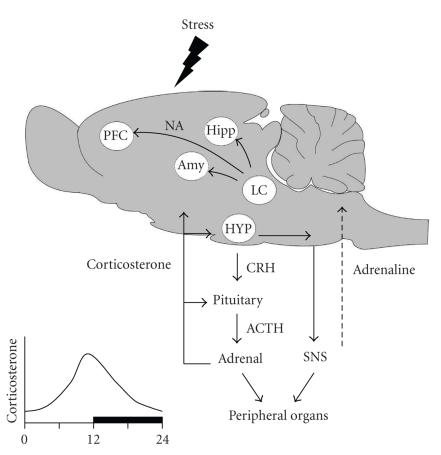
Exposure of a rat to stress may activate many brain regions (depending on the type of stressor), including the amygdala (Amy), hippocampus (Hipp), and prefrontal cortex (PFC). The output of these areas funnels through the hypothalamus (HYP) and there leads to the activation of the fast acting sympatho-adrenomedullar system (right) and the slower acting hypothalamo-pituitary-adrenal axis (left). Both systems not only affect the function of peripheral organs but also feed back on the brain, via adrenaline and corticosterone, respectively. Adrenaline can, via intermediate steps involving the nucleus tractus solitarius, give rise to central release of noradrenaline (NA) from the locus coeruleus (LC), reaching again among other areas the amygdala, prefrontal cortex, and hippocampus. Corticosterone is distributed throughout the brain but acts only at those sites where receptors are enriched. Inset at lower left: the release of corticosterone displays a diurnal rhythm, peaking just before the onset of the active phase. In rats, this is at the end of the light period; in humans, this is just before awakening. SNS = sympathetic nervous system; ACTH = adrenocorticotropin hormone; CRH = corticotropin releasing hormone.

**Figure 2:**
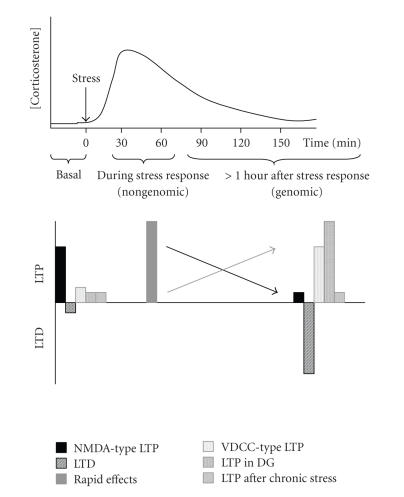
Top: exposure to stress (arrow) leads to a temporary rise in the circulating corticosterone concentration. After approximately two hours, levels are back to the prestress level. Bottom: the consensus view is that exposure to stress reduces NMDA-type LTP in the CA1 area (solid black bar), in a slow gene-mediated fashion. At the same time, LTD is facilitated (striped black bar). Recent studies (greyish bars) have elaborated this view. At the initial phase of the stress response (i.e., as long as corticosteroid levels are really elevated) LTP is increased (solid grey bar); this is most likely due to a nongenomic effect of corticosterone, in concert with the effects of CRH and noradrenaline. At a later time scale (when corticosteroid levels have normalized again), VDCC- (as opposed to NMDA-) type of LTP is increased (stippled grey bar). While LTP in the CA1 area is reduced by stress, LTP in the dentate gyrus (DG) can be enhanced (vertical striped grey bar). Chronic stress suppresses LTP, under basal conditions as well as some time after exposure to elevated corticosteroid levels (horizontal striped grey bar). The black arrow indicates the direction of change in LTP as agreed for gene-mediated effects of high doses of corticosterone on NMDA-type LTP in the CA1 area. The grey arrow indicates the direction for changes regarding the dentate gyrus, VDCC-type of LTP, and rapid nongenomic effects.
